# Factors Affecting Family Satisfaction with Inpatient End-of-Life Care

**DOI:** 10.1371/journal.pone.0110860

**Published:** 2014-11-17

**Authors:** Erin Sadler, Brigette Hales, Blair Henry, Wei Xiong, Jeff Myers, Lesia Wynnychuk, Ru Taggar, Daren Heyland, Robert Fowler

**Affiliations:** 1 Department of General Surgery, University of Toronto, Toronto, Ontario, Canada; 2 Quality and Patient Safety, Sunnybrook Health Sciences Centre, Toronto, Ontario, Canada; 3 Bioethics, Sunnybrook Health Sciences Centre, Toronto, Ontario, Canada; 4 Trauma, Emergency and Critical Care Program, Sunnybrook Health Sciences Centre, Toronto, Ontario, Canada; 5 Palliative Care, Sunnybrook Health Sciences Centre, Toronto, Ontario, Canada; 6 Quality and Patient Safety, Chief Nursing and Health Professions Executive, Sunnybrook Health Sciences Centre, Toronto, Ontario, Canada; 7 Department of Critical Care Medicine, Queen's University, Kingston, Ontario, Canada; 8 Department of Critical Care Medicine, Sunnybrook Health Sciences Centre, Toronto, Ontario, Canada; Department of Medicine, University of Toronto, Toronto, Ontario Canada; Shanghai Mental Health Center, Shanghai Jiao Tong University School of Medicine, China

## Abstract

**Background:**

Little data exists addressing satisfaction with end-of-life care among hospitalized patients, as they and their family members are systematically excluded from routine satisfaction surveys. It is imperative that we closely examine patient and institution factors associated with quality end-of-life care and determine high-priority target areas for quality improvement.

**Methods:**

Between September 1, 2010 and January 1, 2012 the Canadian Health care Evaluation Project (CANHELP) Bereavement Questionnaire was mailed to the next-of-kin of recently deceased inpatients to seek factors associated with satisfaction with end-of-life care. The primary outcome was the global rating of satisfaction. Secondary outcomes included rates of actual versus preferred location of death, associations between demographic factors and global satisfaction, and identification of targets for quality improvement.

**Results:**

Response rate was 33% among 275 valid addresses. Overall, 67.4% of respondents were very or completely satisfied with the overall quality of care their relative received. However, 71.4% of respondents who thought their relative did not die in their preferred location favoured an out-of-hospital location of death. A common location of death was the intensive care unit (45.7%); however, this was not the preferred location of death for 47.6% of such patients. Multivariate Poisson regression analysis showed respondents who believed their relative died in their preferred location were 1.7 times more likely to be satisfied with the end-of-life care that was provided (p = 0.001). Items identified as high-priority targets for improvement included: relationships with, and characteristics of health care professionals; illness management; communication; and end-of-life decision-making.

**Interpretation:**

Nearly three-quarters of recently deceased inpatients would have preferred an out-of-hospital death. Intensive care units were a common, but not preferred, location of in-hospital deaths. Family satisfaction with end-of-life care was strongly associated with their relative dying in their preferred location. Improved communication regarding end-of-life care preferences should be a high-priority quality improvement target.

## Introduction

Most elderly Canadians, when asked, would prefer to die at home. However in 1997, 73% of Canadians spent their final days in hospital and more recent reports estimate between 54–63% of all deaths continue to occur in hospital [1]. Despite most patients desiring a non-technologically assisted death, nearly one-fifth of all hospitalized patients were admitted to an intensive care unit (ICU) on their final hospitalization [2]. Therefore, it is imperative that our health care system evolve both a capacity to provide quality end-of-life (EOL) care in the location preferred by patients; and in parallel, that our health care system comprises clinicians who have the skills to provide compassionate, and high quality EOL care regardless of location of death.

Despite recognizing EOL care as an important area for quality improvement in hospitals, information related to its provision remains limited and insufficient [3]. Most hospitals systematically exclude the names of recently deceased patients and their family members from routine surveys intended to measure satisfaction with care. In 2000, the Canadian Senate recommended federal and provincial health care data custodians develop indicators for EOL care; however, this has yet to be achieved [4]. In Canada, we urgently require information about current practice and performance in order to guide quality improvement in EOL care. Preferences for care at the end of life vary among the population. Therefore, it is important to seek the perspective of patients and families in any assessment of performance. Prior work has demonstrated close patient proxies can reliably report on quality of services and observable symptoms for dying patients [5]. As the Canadian population is increasingly multicultural it is also important that hospitals understand how individual and cultural differences may influence patients' perception of the provision of EOL care.

The aim for this study was to evaluate the provision and quality of EOL care from the perspective of bereaved family members of recently deceased patients in a large academic tertiary care Canadian hospital. The specific objectives were: 1) assess family members' satisfaction with EOL care; 2) identify associations between level of satisfaction and patient factors (e.g. circumstances of death including location, preference of location and cultural factors); and 3) identify high-priority targets for quality improvement in the care of dying patients and their family members.

## Methods

### Study Design

The study received approval from Sunnybrook Health Sciences Centre's Research Ethics Board. Informed consent was obtained from all participants prior to entering the study. This study involved a non-incentivized routine mail-out questionnaire protocol. The Canadian Health care Evaluation Project (CANHELP) Bereavement Questionnaire, a letter of information, and return envelope were post-mailed to a random sample of 352 family member contacts of patients for whom death occurred between September 1, 2010 and January 1, 2012. Among those who had not responded after the first mail-out, a second mail-out was sent just over three weeks later. Although previous studies have demonstrated increased response rates using repeat mail-outs and reminders, the decision were made to limit the number of mail-outs to two in order to limit possible distress among family members [6]. Mail, email and phone contacts were provided in the letter of information to all potential respondents should they wish to contact the study investigators. Given the nature of the population, a sample size was not calculated as the number of participants would be determined by the total number of deaths occurring among admitted patients during the study period and for who contact information of their family member was available.

### Survey Tool

The Canadian Health care Evaluation Project (CANHELP) Bereavement Questionnaire is a 40-item tool that has been previously validated for use in evaluating quality EOL care [7]. Each item has a 5-point Likert scale to rate level of satisfaction [1-not at all satisfied, 2-not very satisfied, 3-somewhat satisfied, 4-very satisfied, 5-completely satisfied]. The items collectively address eight domains of care including patient pain and symptom management; timely and clear communication; information to prepare the family for approaching death; compassionate care, comfort, dignity, and respect; patient-centred decision making; care of the family; family support; and caregiver satisfaction with hospital facilities and staff. At the end of the questionnaire additional optional items regarding ethnicity, language, and spiritual faith were added to explore their respective associations with EOL care satisfaction.

### Data Analysis

The primary outcome variable was the CANHELP Bereavement Questionnaire's global rating of satisfaction which asked: “In general, how satisfied were you with the quality of care your relative received in the last month of life?” Secondary outcomes included: rates of actual versus preferred location of death; associations among demographic factors (patient ethnicity, primary language of patient, religion, and religiosity) and global satisfaction; and identification of targets for quality of care improvement.

Descriptive statistics of demographic data and Likert scale responses included counts and proportions, means (and standard deviations), and medians (and interquartile ranges). The distributions of all responses were assessed visually to determine appropriate parametric or non-parametric analyses. Univariate analysis included Pearson χ^2^ and the Mann-Whitney U Test which examined associations between levels of satisfaction and particular characteristics of the respondents and their deceased relative. Levels of satisfaction were dichotomized into satisfied (“very satisfied or completely satisfied”) vs. not satisfied (“not at all satisfied, not very satisfied, or somewhat satisfied”); ethnicity into Caucasian vs. non-Caucasian; religion into Christian vs. non-Christian; religiosity into religious vs. non-religious; and primary patient language into English speaking vs. other.

Given the high prevalence of the outcome variable “global rating of satisfaction,” the statistic measure of prevalence ratio (relative risk) is preferable to odds ratio. A recent study has proposed a modified Poisson approach to analyze binary data for relative risk with robust error variance [8]. Six independent variables were pre-selected to build a multivariate Poisson regression model. These independent variables were selected *a priori* based on preliminary data exploration and clinical relevance based on the experimental question. A univariate Poisson regression model was applied to each independent variable to identify trending of satisfaction. Age factor was highly insignificant (p = 0.999) in the univariate Poisson regression and thus excluded from the multivariate Poisson regression. The other five independent variables were chosen for multivariate modeling due to significant trending (p<0.2) or clinical relevance. These variables included location of death (ICU vs. other), preferred location of death (yes vs. no), religiosity (religious vs. non-religious), patient language (English-speaking vs. other) and length of stay (7 to17 days vs. 1 to 6 days, or greater than 18 days vs. 1 to 6 days).

A respondent versus non-respondent analysis was performed to assess for significant differences between these population samples. Fisher's Exact test was used to determine significance when cell sizes were less than five. A Missing Value Analysis was performed to identify questions with a high proportion of unanswered values and estimate their potential impact on results [9].

Spearman Correlations were used to define the relative importance of the questionnaire's items by their association with the global rating of satisfaction. Importance-Satisfaction plots were constructed to identify areas that are of high-priority as targets for improvement by plotting the percent of “very” and “completely satisfied” responses (by their Spearman correlation coefficient) with the global rating of satisfaction [7], [10], [11]. Four quadrants were then established by plotting a vertical and horizontal line at the median values of the satisfied responses, and the correlation coefficients, respectively. Items in quadrant A (upper left) were considered to be areas of highest-priority for improvement because they were correlated strongly with global satisfaction, yet had lower ratings of satisfaction. A threshold significance level of α<0.05 was used for all analyses. All statistical analysis was completed using SPSS 20.0 (IBM Corporation, Armonk, NY) or SAS 9.3 (SAS Institute, Cary NC).

## Results

Response rate was 33% (92/275 valid addresses from an initial sample of 352 deceased patients) after two mail-outs (57 responses after the first, 35 after the second). The original sample of 352 was reduced to 275 valid addresses due to questionnaire packages that were returned to sender. Missing Value Analyses revealed that non-responses to items were missing randomly as opposed to systematically (Little MCAR test, p = 0.820). Only one respondent elected not to answer the optional demographic and cultural items. There were no significant differences among respondents and non-respondents according to age of the deceased, cause of death of the deceased, location of death, length of stay, duration of time since death, or relationship with the deceased; however family member contacts of deceased male patients were more likely to respond than family member contacts of deceased female patients (OR 1.77, p = 0.028) (Table 1).

**Table 1:** Characteristics of Respondents and Non-Respondents.

	Respondents Mean (SD) or N (%)	Non-Respondents Mean (SD) or N (%)	χ^2^	p
N	92 (33.5%)	183 (67%)	-	
Age (years)	75.7 (16.0)	74.2 (17.8)	-	0.500
*Sex of patient*			4.81	0.028
Male	58 (63.7%)	91 (49.7%)	-	
Female responses	45 (77.6%)			
Male responses	13 (22.4%)			
Female	33 (36.3%)	92 (50.3%)	-	
Female responses	11 (33.3%)			
Male responses	22 (66.7%)			
Length of stay (days)	15.5 (30.1)	16.9 (33.6)		0.742
Time since death (days)	41.8 (22.0)	46.4 (22.1)		0.112
*Location of death*				0.583
Intensive care unit	41 (45.1%)	77 (42.1%)		
Hospital ward	48 (52.7%)	95 (51.9%)		
Other	2 (2.2%)	11 (6.0%)		
*Relationship to patient*			2.11	0.715
Spouse	52 (57.1%)	98 (53.6%)		
Son/Daughter	29 (31.9%)	69 (37.7%)		
Sibling	3 (3.2%)	3 (1.6%)		
Parent	2 (2.2%)	6 (3.3%)		
Other	5 (5.5%)	7 (3.8%)		
*Cause of death*			11.19	0.263
Cancer	21 (23.1%	54 (39.5%)		
Cardiac	19 (20.8%)	28 (15.3%)		
Respiratory	16 (17.6%)	24 (13.1%)		
Neurological	14 (15.4%)	16 (8.7%)		
Trauma	7 (7.7%)	17 (9.3%)		
Sepsis	6 (6.6%)	10 (5.5%)		
Other	4 (4.4%)	12 (6.6%)		
Vascular	3 (3.3%)	8 (4.4%)		
Renal	0	5 (2.7%)		
GI	1 (1.1%)	9 (4.9%)		

Abbreviations: GI =  gastrointestinal.

Overall, 67.4% of respondents were *very* or *completely* satisfied with the overall quality of care their relative received in the last month of life. The most common location of death was the ICU (45.7%); however, this was not the preferred location of death for 47.6% of patients dying in the ICU (Table 2). Overall, 46.7% believed their relative did not die in the preferred location and of these, 71.4% believed their family member would have preferred an out-of-hospital (“home or retirement home”) location. In Table 3, univariate Poisson regression analysis showed that, respondents who believed their relative died in their preferred location of death were 1.9 times more likely to be satisfied with the quality of EOL care (p*<*0.001).

**Table 2:** Actual and Preferred Location of Death.

Location of Death	N (%)
Intensive Care Unit	42 (45.7)
Palliative Care Unit	4 (4.3)
Hospital Ward	39 (42.4)
Other	3 (3.3)
No Answer	4 (4.3)
*Patient's Preferred Location*	
Yes	38 (41.3)
No	42 (45.7)
Prefer Home or Retirement Home	30 (71.4)
Prefer Other	12 (28.6)
No Answer	12 (13)

**Table 3:** Factors Associated with Satisfaction with End-of-Life Care.

Variables	Categories	Univariate Poisson regression		Multivariable Poisson regression	
		Prevalence ratio (95% CI)	p value	Prevalence ratio (95% CI)	p value
Preferred Location of Death	Yes vs No	1.9 (1.4–2.8)	<0.001	1.7 (1.2–2.4)	0.001
Location of Death	ICU vs Other	1.2 (0.9–1.6)	0.279	1.2 (0.9–1.6)	0.120
Religiosity	Very or Somewhat vs Other	1.5 (1.0–2.1)	0.020	1.2 (0.9–1.7)	0.191
Language	English vs Other	0.7 (0.5–0.9)	0.006	0.9 (0.7–1.1)	0.316
Age	> = 80	1.0 (0.6–1.7)			
	71–79		0.999		
	< = 70 (ref)	1.0 (0.7–1.4)			
Length of Stay	> = 18	0.9 (0.7–1.2)		0.9 (0.7–1.2)	
	7–17		0.133		0.744
	< = 6 (ref)	0.6 (0.4–1.0)		0.8 (0.5–1.4)	

Abbreviations: ref  =  reference; vs  =  versus.

The risk-adjusted multivariate Poisson regression model also indicated that patients' preferred location was a highly significant predictor for family global satisfaction (p = 0.001; Table 3). Families of patients who died in their preferred location were 1.7 times more likely to be satisfied with the quality of EOL care than families of patients who did not die in their preferred location using the multivariate model. The other four variables (location of death, religiosity, patient language and length of stay) did not demonstrate a significant relationship with family global satisfaction. The multivariate Poisson model fits the data well, since the ratio of deviance over degrees of freedom was less than 1.0, suggesting that there was no over-dispersion in the data. A Poisson model may be preferable over negative binomial model when the data is not over-dispersed.

**Figure 1:**
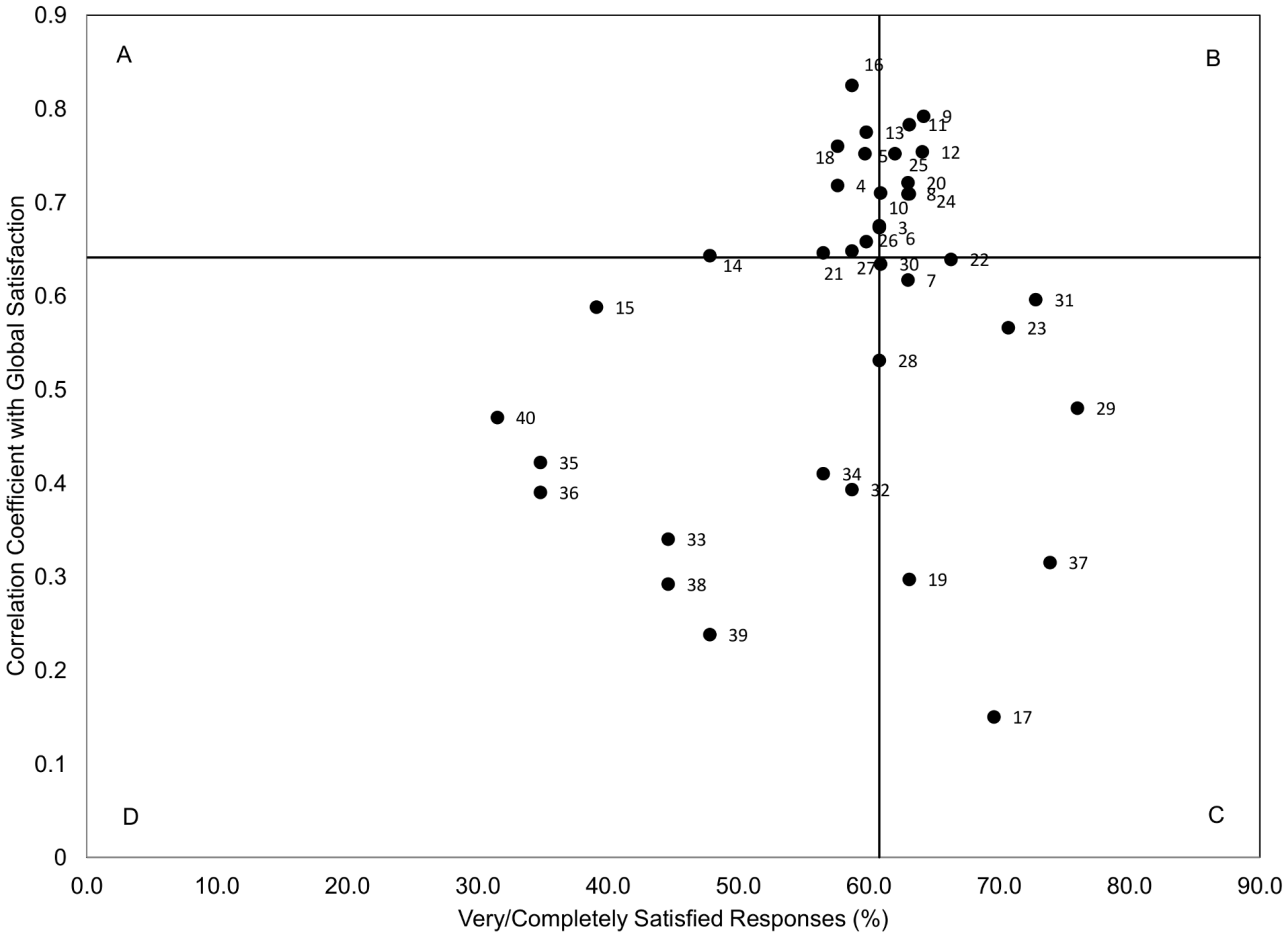
Importance Satisfaction Grid.

Figure 1 outlines the Importance-Satisfaction Grid [7], [10], [11]. Quadrant A identifies survey items that are high-priority targets for improvement and include: relationships with the doctors; characteristics of the doctors and nurses; illness management; and communication and decision-making (Table 4). The survey items in quadrant D signify areas less correlated with global satisfaction, but are potentially worthwhile targets given the lower levels of satisfaction with current practice.

**Table 4:** High-Priority Areas for Quality Improvement.

Domain		Questionnaire Item
**Relationship with Doctors**	3.	How satisfied are you that you knew the doctor(s) in charge of your relative's care in the last month of life?
	4.	How satisfied are you that the doctor(s) took a personal interest in your relative in the last month of life?
	5.	How satisfied are you that the doctor(s) were available when you or your relative needed them (by phone or in person) in the last month of life?
	6.	How satisfied are you with the level of trust and confidence you had in the doctor(s) who looked after your relative in the last month of life?
**Characteristics of the Doctors and Nurses**	10.	How satisfied are you that the doctors and nurses looking after your relative in the last month of life were compassionate and supportive of you.
**Illness Management**	13.	How satisfied are you that physical symptoms (i.e. pain, shortness of breath, nausea, etc.) your relative had in the last month of life were adequately assessed and controlled?
	16.	How satisfied are you that, in the last month of life, your relative received good care when you were not able to be with him/her?
	18.	How satisfied are you that health care workers worked together as a team to look after your relative in the last month of life?
	21.	How satisfied are you that care and treatment your relative received in the last month of life was consistent with his/her wishes?
**Communication and Decision Making**	26.	How satisfied are you that the doctor(s) listened to what you had to say in the last month of your relative's life?
	27.	How satisfied are you with the discussions in the last month of life with the doctor(s) about where your relative would be cared for (in hospital, home, elsewhere)?

## Discussion

Although family members of recently deceased patients were generally satisfied with the quality of EOL care, this study identified several important targets for quality improvement. First, nearly half of respondents believed that patients did not die in their preferred location, and most commonly would have preferred an out-of-hospital, at home death. Second, despite the population stating that they would like to avoid aggressive, technology-laden EOL care, the most common location of death in hospital was the ICU. Importantly, families of patients who died in their preferred location were nearly two times more likely to be satisfied with EOL care.

Although only one of many factors, dying in a preferred location has been shown in other studies to be strongly associated with satisfaction [12]. This indicates that our health care system should evolve mechanisms to provide such care outside of hospitals, in palliative homes or hospices; and, hospitals should reassess resources available to provide EOL care in alternative settings to the ICU. Our findings are consistent with the work of others who have found a mismatch in the care which patients and their families' desire, and the default institutionalized and aggressive EOL care which they often receive [2].

The discrepancy between preferred and actual location of death might also be due to ineffective advance care planning processes. In a recently performed study of 513 elderly inpatients at high risk of dying at 12 Canadian acute care hospitals, although 76% of patients had thought about EOL care, only 12% had preferred life-prolonging care, and fewer than half had completed an advance care plan. Of those who had discussed their wishes, 30% had done so with their family physician and only 55% with any member of the health care team [13]. Before hospitalization, only 20% of patients discussed prognosis with a physician.

The Importance-Satisfaction grid highlights the areas of EOL care that should be high-priority targets for quality of care improvement. Our results echo those of other investigators who surveyed both patients and families finding that emotional support for patients, relationships with doctors, and communication and decision-making are highly correlated with satisfaction yet often poorly performed [7], [14]. Items most strongly correlated with global satisfaction included the level of trust in the doctor(s) and nurse(s), availability of the doctor(s) when their relative needed them, and compassionate and supportive care of their relative and themselves. One systematic review highlighted several similar areas of EOL care that were important to overall satisfaction, including accessibility and coordination, effective symptom management, comfort level with the dying process, education and communication, emotional support, care on the individual level, and support of patients' decision-making [15]. The similarity with other literature adds confidence to this study's findings and lends support to generalizability to other care settings. Our results suggest that levels of satisfaction with the quality of EOL care may be improved by targeting a select number of high-impact aspects of care, such as doctor availability, caring for patients in the absence of family members, the health care team working together as a cohesive team, and attentive listening to patients and families.

An overarching and important finding from this study is that the CANHELP Bereavement Questionnaire provides a feasible tool to evaluate care provided to patients at the end of life, to measure satisfaction, and to identify high-priority targets for quality improvement. This has been shown in other literature to be a reasonable method of obtaining accurate and representative views of the patients [5]. In addition, surveying family members can be a powerful vehicle for families to not only provide feedback, but to share their experiences, which may be an important element of the grieving process. A substantial number of returned surveys included narratives of the experience. Many requested an opportunity to communicate with study investigators directly, and this provided a subsequent mechanism to support families through their loss, and, possibly an additional mechanism to improve their own satisfaction with care delivery.

This study is not without limitations. First, the response rate to the survey was modest. The response rate may have improved with subsequent mail-outs; however, we purposely limited such reminders in case families were not yet ready or willing to engage in discussion of their recent loss. As well, there has been a recent shift towards assessing survey response rate by the potential for non-respondent bias, and whether the response population is representative of the population of interest [16]. Importantly, we found very little difference in measured characteristics of respondents and non-respondents. However, this modest response rate does limit the external validity of the current study, and it is recommended that larger datasets be collected in order to determine reliability of the present results. Second, the respondent population was predominantly Caucasian, English-speaking and Christian. Ensuring delivery of culturally sensitive care should be an important goal for hospitals as we have previously shown that such factors often predict desires for different care at the end of life [17]. Although our respondent pool was of only modest size, it is important that we did not find evidence that specific aspects of culture – ethnicity, language, religion – were significantly associated with satisfaction with the quality of EOL care in this setting. It is possible that in more or less culturally diverse patient settings, such factors may have an even greater relationship with satisfaction. A related limitation is that our survey was administered only in English. The questionnaire is also available in French, and future work should explore further translations, especially among ethnic groups that have may have culturally unique EOL needs or practices. An additional limitation to the dataset is the anonymized nature of the survey responses. Analysis of the demographic data of the respondents and non-respondents was done on a pooled, gross level. However, respective survey responses were anonymized from their demographic data in order to fulfill the Research Ethics requirements of the study's institution. This meant that analysis at the individual respondent level was unable to be completed. Finally, although the results of our study represent the views of families at a single tertiary academic centre, we suspect the emerging of importance of communication, relationships and location of death are broadly generalizable to other hospitals and we encourage other centres to undertake a similar exercise towards understanding how to better improve quality of EOL care.

## Conclusion

Administration of the CANHELP bereavement questionnaire to family members of recently deceased inpatients is feasible and associated with a high degree of internal consistency. Many patients die in a location other than is preferred and dying in the preferred location is highly associated with family satisfaction with EOL care. This represents an urgent health care system and hospital target for quality improvement. Additionally, this questionnaire allows construction of a hospital-specific Importance-Satisfaction grid that can help hospitals identify unique quality improvement targets. We recommend that hospitals and health care systems adopt a common instrument to measure and improve EOL care that allows for local quality of care improvement and comparisons across institutions en route to addressing the needs of patients and families at the end of life.
